# Metabolic phenotyping reveals a reduction in the bioavailability of serotonin and kynurenine pathway metabolites in both the urine and serum of individuals living with Alzheimer’s disease

**DOI:** 10.1186/s13195-020-00741-z

**Published:** 2021-01-09

**Authors:** Luke Whiley, Katie E. Chappell, Ellie D’Hondt, Matthew R. Lewis, Beatriz Jiménez, Stuart G. Snowden, Hilkka Soininen, Iwona Kłoszewska, Patrizia Mecocci, Magda Tsolaki, Bruno Vellas, Jonathan R. Swann, Abdul Hye, Simon Lovestone, Cristina Legido-Quigley, Elaine Holmes, on behalf of AddNeuroMed consortium, Hilkka Soininen, Iwona Kłoszewska, Patrizia Mecocci, Magda Tsolaki, Bruno Vellas, Simon Lovestone

**Affiliations:** 1 UK Dementia Research Institute, Imperial College London, Hammersmith Hospital, London, W12 0NN UK; 2 Health Futures Institute, Murdoch University, Perth, WA 6105 Australia; 3 The Perron Institute for Neurological and Translational Science, Nedlands, WA 6009 Australia; 4 Section of Bioanalytical Chemistry W12 0NN, UK, Imperial College London, Hammersmith Hospital, London, W12 0NN UK; 5 National Phenome Centre, Imperial College London, Hammersmith Hospital, London, W12 0NN UK; 6 imec, Exascience Life Lab, Kapeldreef 75, B-3001 Leuven, Belgium; 7 Institute of Psychiatry, Psychology and Neuroscience, King’s College London, London, UK; 8 Present address: Core Metabolomics and Lipidomics Laboratory, Metabolic Research Laboratories, Institute of Metabolic Science, University of Cambridge, Cambridge, CB2 0QQ UK; 9 Department of Neurology, University of Eastern Finland and Kuopio University Hospital, Kuopio, Finland; 10 Medical University of Lodz, Lodz, Poland; 11 Institute of Gerontology and Geriatrics, University of Perugia, Perugia, Italy; 12 3rd Department of Neurology, Aristotle University, Thessaloniki, Greece; 13 INSERM U 558, University of Toulouse, Toulouse, France; 14 Department of Psychiatry, Warneford Hospital, University of Oxford, Oxford, UK; 15 Current affiliation at Janssen-Cilag Ltd, High Wycombe, UK; 16 Steno Diabetes Center Copenhagen, Gentofte, Denmark; 17 Section for Nutrition Research, Imperial College, Hammersmith Campus Du Cane Road, London, W12 0NN UK

**Keywords:** Alzheimer’s disease, Kynurenine, Tryptophan, Serotonin, Metabolic phenotyping, Mass spectrometry, Systemic inflammation, Serotonergic signalling

## Abstract

**Background:**

Both serotonergic signalling disruption and systemic inflammation have been associated with the pathogenesis of Alzheimer’s disease (AD). The common denominator linking the two is the catabolism of the essential amino acid, tryptophan. Metabolism via tryptophan hydroxylase results in serotonin synthesis, whilst metabolism via indoleamine 2,3-dioxygenase (IDO) results in kynurenine and its downstream derivatives. IDO is reported to be activated in times of host systemic inflammation and therefore is thought to influence both pathways. To investigate metabolic alterations in AD, a large-scale metabolic phenotyping study was conducted on both urine and serum samples collected from a multi-centre clinical cohort, consisting of individuals clinically diagnosed with AD, mild cognitive impairment (MCI) and age-matched controls.

**Methods:**

Metabolic phenotyping was applied to both urine (*n* = 560) and serum (*n* = 354) from the European-wide AddNeuroMed/Dementia Case Register (DCR) biobank repositories. Metabolite data were subsequently interrogated for inter-group differences; influence of gender and age; comparisons between two subgroups of MCI - versus those who remained cognitively stable at follow-up visits (sMCI); and those who underwent further cognitive decline (cMCI); and the impact of selective serotonin reuptake inhibitor (SSRI) medication on metabolite concentrations.

**Results:**

Results revealed significantly lower metabolite concentrations of tryptophan pathway metabolites in the AD group: serotonin (urine, serum), 5-hydroxyindoleacetic acid (urine), kynurenine (serum), kynurenic acid (urine), tryptophan (urine, serum), xanthurenic acid (urine, serum), and kynurenine/tryptophan ratio (urine). For each listed metabolite, a decreasing trend in concentrations was observed in-line with clinical diagnosis: control > MCI > AD. There were no significant differences in the two MCI subgroups whilst SSRI medication status influenced observations in serum, but not urine.

**Conclusions:**

Urine and serum serotonin concentrations were found to be significantly lower in AD compared with controls, suggesting the bioavailability of the neurotransmitter may be altered in the disease. A significant increase in the kynurenine/tryptophan ratio suggests that this may be a result of a shift to the kynurenine metabolic route due to increased IDO activity, potentially as a result of systemic inflammation. Modulation of the pathways could help improve serotonin bioavailability and signalling in AD patients.

**Supplementary Information:**

The online version contains supplementary material available at 10.1186/s13195-020-00741-z.

## Background

The pathogenesis of Alzheimer’s disease (AD) has previously been associated with both systemic inflammation [1, 2] and disruption of the serotonergic signalling system [3–5]. Metabolically, a common link between the two biological processes is the catabolism of the essential amino acid tryptophan. Despite its primarily use in protein synthesis [6], tryptophan also undergoes enzymatic conversion via two distinct metabolic pathways. The majority of free tryptophan is metabolised via the enzymes tryptophan 2,3-dioxygenase (TDO) and indoleamine 2,3-dioxygenase (IDO) in the kynurenine pathway. This results in both neuroactive (kynurenic acid, 3-hydroxykynurenine [7]) and neurotoxic (quinolinic acid [8]) molecules that can influence the central nervous system. Metabolites originating from this metabolic route have been reported to associate with both AD disease status [9] and levels of the AD-affiliated proteins, amyloid-β and neurofilament light chain [10–12].

A secondary metabolic pathway occurs in the enzymes tryptophan hydroxylase and 5-hydroxytryptophan decarboxylase leading to the production of the key neurotransmitter serotonin [13]. Serotonin homeostasis has been linked to AD, and disruptions in serotonergic signalling are reported to enhance amyloid-β pathology in vitro [5], in vivo [4] and in human clinical studies [3, 4]. Previous literature suggests the mechanistic route for this influence is a result of serotonin receptor activation upregulating α-secretase activity, shifting the cleavage of amyloid precursor protein away from the β- and γ- secretase route and reducing amyloid-β production [5]. Indeed, serotonin signalling has been a research target of therapeutic intervention in the disease, with the ongoing evaluation of selective serotonin reuptake inhibitor (SSRI) useage to increase the bioavailability of serotonin at nerve terminals, and therefore to attempt to control AD symptoms and cognitive decline [14, 15].

The metabolic balance between both pathways, and therefore the subsequent bioavailability of downstream metabolites, is reported to be influenced by the homeostatic control of the IDO enzyme [13]. In times of host systemic inflammation, IDO is upregulated by circulating cytokines, thereby increasing the metabolic turnover of tryptophan to kynurenine. As such, the kynurenine/tryptophan ratio has been reported to be a biomarker for detecting systemic inflammation in disease [16, 17].

Due to the links between AD pathogenesis, systemic inflammation and serotonergic signalling, the two pathways have previously been investigated in the disease, with changes in the concentration of circulating metabolites from both pathways reported in individuals clinically diagnosed with AD compared with controls. However, such investigations have been typically limited to small pilot studies and have not covered the full range of metabolites involved in the two pathways [9, 10, 18, 19].

Biological pathways of interest that are implicated in health and disease can be effectively investigated using a technique known as metabolic phenotyping. Frequently, the technique is now being applied to large epidemiological and clinical cohorts to investigate metabolic changes that influence population health and disease [20]. Such application of discovery-based metabolic phenotyping in clinical cohort studies of AD has previously reported differences in the metabolism of lipids [21, 22], fatty acids [23] and amino acids [24], but few studies have used the technology to annotate and target specific pathways of interest in the disease.

**Fig. 1:**
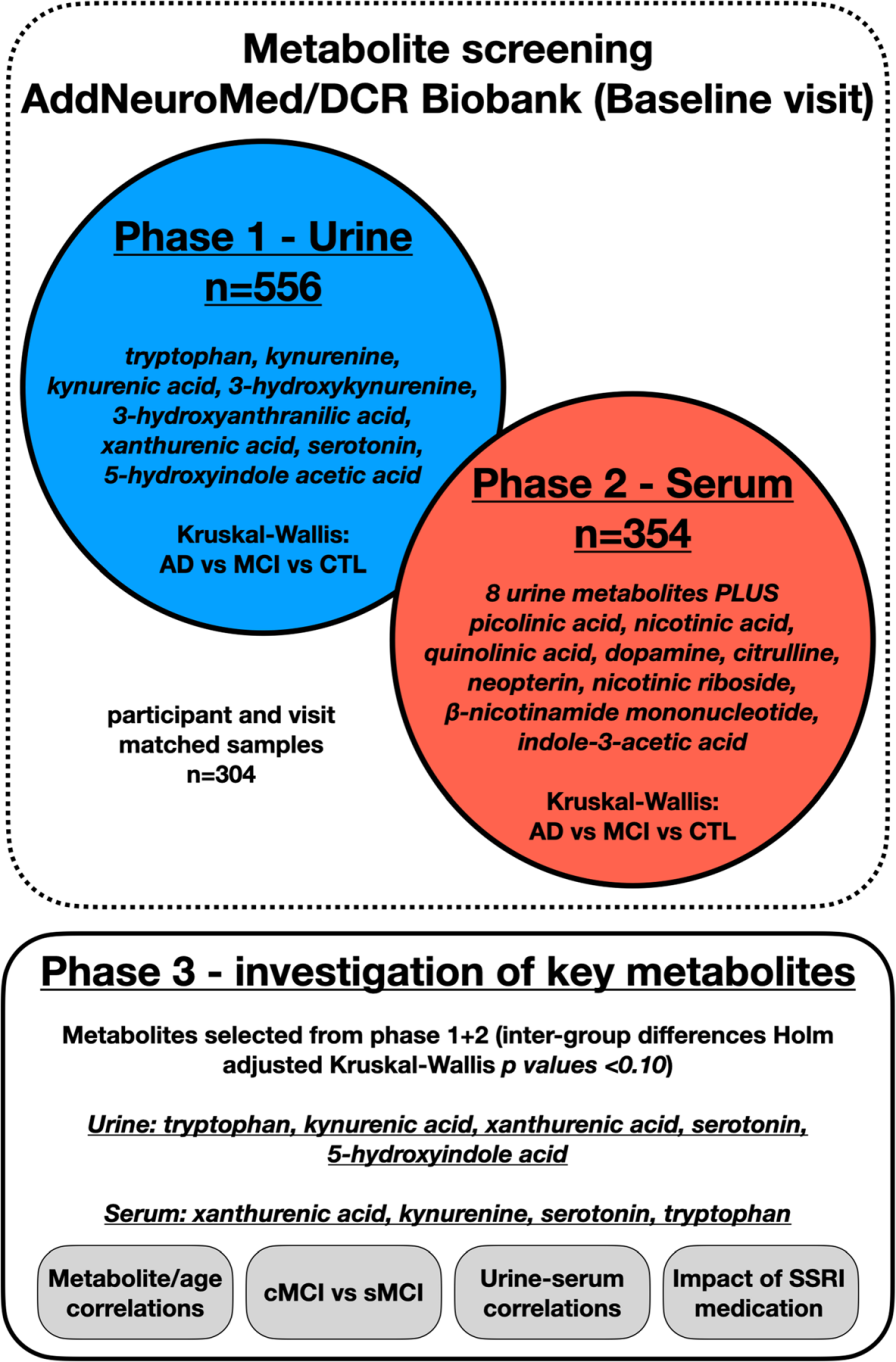
Study workflow. An overview of the study design and overall workflow

Here, a multi-stage metabolic phenotyping study (Fig. 1), was employed to investigate urinary and serum levels of tryptophan and its metabolites in a cohort of participants diagnosed with AD, MCI and age-matched controls. Initially, metabolite profiling was used as an introductory screening technique to investigate eight metabolites in urine (Table 3), before applying a targeted analysis of the tryptophan pathway in serum to ascertain if metabolite differences were reflected in the circulatory system.

## Methods

### Participants

Study participants were from the European AddNeuroMed and the London based Dementia Case Register (DCR) projects [25–27]. Participants aged between 57 and 97 were recruited either as controls reporting normal cognition or with a clinical diagnosis of Alzheimer’s disease (AD) or mild cognitive impairment (MCI). The assessment protocol has been previously described [25, 26, 28], but in brief, the protocol was based on a clinical assessment including a structured clinical interview together with cognitive assessments including ADAS-COG, MMSE and CERAD-NB with a final diagnosis being made according to NINCDS-ADRDA and DSM-IV criteria.

Within the MCI group, cognition was monitored at follow-up visits—those who remained cognitively stable at follow-up visits were classed as stable MCI (sMCI), whilst the second group experienced cognitive decline and received a later diagnosis of AD and were classed as converting MCI (cMCI).

Serum and urine samples were collected and stored frozen in aliquots at − 80 °C until use. The samples were from baseline collections only and had not been subjected to any freeze-thaw cycles. Mass spectrometry analysis was completed on 556 urine samples. Where available, matched serum samples were then analysed, resulting in the generation of spectra from 354 serum samples. An overview of study samples can be seen in Table 1.

**Table 1:** Participant overview

	Total cohort	Control	MCI	AD
**Urine**				
Participants	556	171	209	176
Male/female	269/287	83/88	95/114	91/85
Mean age (SD)	76.24 (5.76)	75.85 (5.17)	76.33 (6.03)	76.53 (5.99)
MMSE score	25.67 (4.59)	28.73 (1.92)	26.86 (2.75)	21.17 (4.87)
CDR	0.56 (0.51)	0.07 (0.18)	0.49 (0.08)	1.10 (0.54)
Reported SSRI medication	43	4	16	23
**Serum**				
Participants	354	86	165	103
Male/female	165/189	44/42	71/94	50/53
Mean age	76.95 (6.13)	75.97 (5.67)	77.50 (6.49)	76.91 (5.84)
MMSE score	25.57 (4.37)	28.80 (1.99)	26.73 (2.24)	21.06 (4.79)
CDR	0.59 (0.54)	0.04 (0.16)	0.49 (0.11)	1.16 (0.55)
Reported SSRI medication	30	1	11	18

Overview of the sample cohort used in the study. *SSRI* selective serotonin reuptake inhibitor, *MCI* mild cognitive impairment, *AD* Alzheimer’s disease, *MMSE* Mini-Mental State Examination score, *CDR* Clinical Dementia Rating

### Study phase 1—Metabolic phenotyping of tryptophan pathway metabolites in urine

Urine samples (*n* = 556) underwent metabolite profiling using an ultra-high-performance liquid chromatography system coupled to a high-resolution quadrupole-time-of-flight mass spectrometer (UHPLC-QTOF-MS) accordig to a previously published method [29]. Eight metabolic features were then annotated (xanthurenic acid, kynurenic acid, serotonin, 5-hydroxyindoleacetic acid, tryptophan, 3-hydroxyanthranilic acid, 3-hydroxykynurenine, kynurenine), integrated and normalised for dilution using quantitative creatinine values derived by a previously published proton nuclear magnetic resonance (^1^H-NMR) method [30]. All methods are described in detail in supplementary information.

Annotated data outputs (exported in comma-separated value format) were imported into R (v.3.5.2) for statistical analysis. Samples that were greater than five standard deviations above the mean for each metabolite were removed as outliers. Shapiro-Wilk testing demonstrated that the metabolite data were not normally distributed (*p* < 0.05). Therefore, non-parametric Kruskal-Wallis tests were performed. Final *p* values were adjusted for multiple testing using the method described by Holm [31]. For metabolites with a Holm-adjusted *p* value of < 0.1, post hoc Dunn’s tests were completed on each pair of participant groups in order to report statistically significant differences between each of the three participant groups (AD, MCI and age-matched controls).

### Study phase 2—Quantification of tryptophan pathway metabolites in serum

In phase 2 of the study, 17 metabolites (xanthurenic acid, kynurenine, serotonin, tryptophan, 3-hydroxyanthranilic acid, kynurenic acid, 3-hydroxykynurenine, β-nicotinamide mononucleotide, picolinic acid, 5-hydroxyindoleacetic acid, nicotinic acid, quinolinic acid, dopamine, neopterin, nicotinic riboside, citrulline, indole-3-acetic acid) were fully quantified, and an additional metabolite (NAD^+^) was semi-quantified in serum using a previously validated UHPLC-tandem mass spectrometry (UHPLC-MS/MS) method [32]. The method is described further in supplementary information.

Statistical analysis was completed in R (v.3.5.2). Again, samples that were greater than five standard deviations above the mean for each metabolite were removed as outliers. Shapiro-Wilk testing demonstrated that the serum metabolite data were not normally distributed (*p* < 0.05); therefore, non-parametric Kruskal-Wallis tests were performed. Final *p* values were adjusted for multiple testing using the method described by Holm [31], and again, metabolites with a Holm-adjusted *p* value of < 0.1 underwent post hoc Dunn’s tests to identify statistically significant differences between each of the three participant groups (AD, MCI and age-matched controls).

### Study phase 3—Further investigation of key metabolites

Metabolites that reported a Holm-adjusted *p* value of < 0.1 from Kruskal-Wallis tests in study phases 1 and 2 were selected for further statistical analysis detailed below.

#### Metabolite correlation with age

Pearson correlation to investigate associations with age.

#### Metabolite comparison between MCI subgroups

Mann-Whitney *U* tests were performed to compare two subgroups of participants who were clinically diagnosed with MCI at the baseline visit. One group remained cognitively stable at subsequent follow-up visits (stable MCI (sMCI)), whilst the second group experienced further cognitive decline and received a diagnosis of AD at subsequent follow-up visits (converting MCI (cMCI)). Details of the cMCI and sMCI groups are displayed in Table 2.

**Table 2:** Mild cognitive impairment participant overview

	Total MCI	sMCI	cMCI
**Urine**			
Participants	209	167	42
Male/female	95/114	80/87	15/27
Mean age (SD)	76.33 (6.03)	76.17 (5.60)	76.91 (7.38)
MMSE	26.86 (2.75)	26.93 (2.94)	26.55 (1.76)
CDR	0.49 (0.08)	0.48 (0.09)	0.51 (0.08)
Reported SSRI medication	16	12	4
**Serum**			
Participants	165	90	75
Male/female	71/94	42/48	29/46
Mean age	77.50 (6.49)	75.98 (5.95)	79.32 (6.67)
MMSE	26.73 (2.24)	26.81 (2.38)	26.63 (2.07)
CDR	0.49 (0.11)	0.48 (0.10)	0.53 (0.12)
Reported SSRI medication	11	8	3

Overview of the two MCI subgroups used in the study. All samples in the study were taken at baseline; however, one MCI subgroup remained stable throughout follow-up visits (sMCI), whilst the second converted to a clinical diagnosis of AD at subsequent follow-up visits (cMCI). *SSRI* selective serotonin reuptake inhibitor, *MMSE* Mini-Mental State Examination score, *CDR* Clinical Dementia Rating

#### Metabolite associations across biofluids

Pearson correlation analysis was performed to investigate the relationship of metabolites across both biofluids (serum and urine). This was performed where matched serum/urine samples from the same participant study visit were available (*n* = 304).

#### Impact of selective serotonin reuptake inhibitor (SSRI) medication

The effect of SSRI medication on the study cohort was investigated by comparing metabolite levels of two AD subgroups: those prescribed SSRI medication vs no SSRI prescribed medication. Data were then re-analysed using Kruskal-Wallis testing as described above, using only study participants who did not report a prescription for SSRI medication.

## Results

### Study phase 1—Metabolic phenotyping of tryptophan pathway metabolites in urine

Good reproducibility was obtained throughout the analysis as determined from the biological QC samples (*n* = 64) analysed across the run, which reported relative standard deviation values for each metabolite ranging from 7.7 to 13.2%.

Kruskal-Wallis testing reported significant inter-group metabolite differences for tryptophan (*p* = 0.0128, Holm-adjusted *p* = 0.0514), serotonin (*p* = 0.0020, Holm-adjusted *p* = 0.0137), xanthurenic acid (*p* = 0.0002, Holm-adjusted *p* = 0.0015), 5-hydroxyindoleacetic acid (*p* = 0.0059, Holm-adjusted *p* = 0.0293), kynurenic acid (*p* = 0.0009, Holm-adjusted *p* = 0.0071) and the kynurenine/tryptophan ratio (*p* = 0.0025, Holm-adjusted *p* = 0.0153) (Table 3).

**Table 3:** Summary of Kruskal-Wallis univariate analysis

Metabolite	***p*** value (Kruskal-Wallis test)	Adjusted ***p*** value (Holm)	Dunn’s post hoc test ***p*** value		
			CTL-AD	CTL-MCI	MCI-AD
**Urine—xanthurenic acid**	**0.0002**	**0.0015**	**0.0001**	**0.0016**	**0.1615**
**Urine—kynurenic acid**	**0.0009**	**0.0071**	**0.0005**	**0.0047**	**0.181**
**Urine—serotonin**	**0.0020**	**0.0137**	**0.0016**	**0.2724**	**0.0047**
**Urine—5-hydroxyindoleacetic acid**	**0.0059**	**0.0293**	**0.0020**	**0.0842**	**0.0517**
**Urine—tryptophan**	**0.0128**	**0.0514**	**0.0050**	**0.098**	**0.0748**
Urine**—**3-hydroxyanthranilic acid	0.0495	0.1484	*NA*	*NA*	*NA*
Urine**—**3-hydroxykynurenine	0.4235	0.8470	*NA*	*NA*	*NA*
Urine**—**kynurenine	0.8738	0.8738	*NA*	*NA*	*NA*
**Urine—kynurenine/tryptophan ratio**	**0.0040**	**0.0242**	**0.0021**	**0.0138**	**0.1966**
**Serum—xanthurenic acid**	**0.0018**	0.0339	**0.0019**	**0.0260**	**0.2796**
**Serum—kynurenine**	**0.0019**	0.0339	**0.0006**	**0.0429**	**0.0286**
**Serum—serotonin**	**0.0037**	0.0625	**0.0016**	**0.0178**	**0.1035**
**Serum—tryptophan**	**0.0047**	0.0756	**0.0024**	**0.0150**	**0.1407**
Serum—3-hydroxyanthranilic acid	0.0222	0.3326	*NA*	*NA*	*NA*
Serum—kynurenic acid	0.0222	0.3326	*NA*	*NA*	*NA*
Serum—3-hydroxykynurenine	0.0236	0.3326	*NA*	*NA*	*NA*
Serum—β-nicotinamide mononucleotide	0.0241	0.3326	*NA*	*NA*	*NA*
Serum—picolinic acid	0.0518	0.5701	*NA*	*NA*	*NA*
Serum—5-hydroxyindoleacetic acid	0.0991	0.9912	*NA*	*NA*	*NA*
Serum—nicotinic acid	0.1066	0.9912	*NA*	*NA*	*NA*
Serum—quinolinic acid	0.1828	1.0000	*NA*	*NA*	*NA*
Serum—NAD+	0.2589	1.0000	*NA*	*NA*	*NA*
Serum—dopamine	0.3926	1.0000	*NA*	*NA*	*NA*
Serum—neopterin	0.4934	1.0000	*NA*	*NA*	*NA*
Serum—nicotinic riboside	0.6746	1.0000	*NA*	*NA*	*NA*
Serum citrulline	0.8351	1.0000	*NA*	*NA*	*NA*
Serum—indole-3-acetic acid	0.8518	1.0000	*NA*	*NA*	*NA*
Serum—kynurenine/tryptophan ratio	0.2507	1.0000	*NA*	*NA*	*NA*

*p* values from Kruskal-Wallis and subsequent post hoc tests using the Holm test to correct for multiple testing. Metabolites with a Holm-adjusted *p* value of < 0.1 are highlighted in bold and underwent a post hoc Dunn’s test to observe differences between subgroups. Metabolites with a Holm-adjusted *p* value of > 0.1 did not undergo a post hoc Dunn’s test and are labelled accordingly with NA

For metabolites that reported a Holm-adjusted *p* value of < 0.1, post hoc Dunn’s tests were performed to investigate differences between the individual study groups. The key urinary metabolites when comparing control to AD were urinary xanthurenic acid (*p* = 0.0001), kynurenic acid (*p* = 0.0005), serotonin (*p* = 0.0016), 5-hydroxyindoleacetic acid (*p* = 0.0020), tryptophan (*p* = 0.0050) and the kynurenine/tryptophan ratio (*p =* 0.0021) (Table 3). Urine metabolite concentrations were also observed to be significantly lower when comparing MCI to the control groups for xanthurenic acid (*p* = 0.0016), kynurenic acid (*p* = 0.0047) and the kynurenine/tryptophan ratio (*p =* 0.0073), whilst serotonin was observed to be lower in AD compared with the MCI group (*p* = 0.0047) (Table 3).

**Fig. 2:**
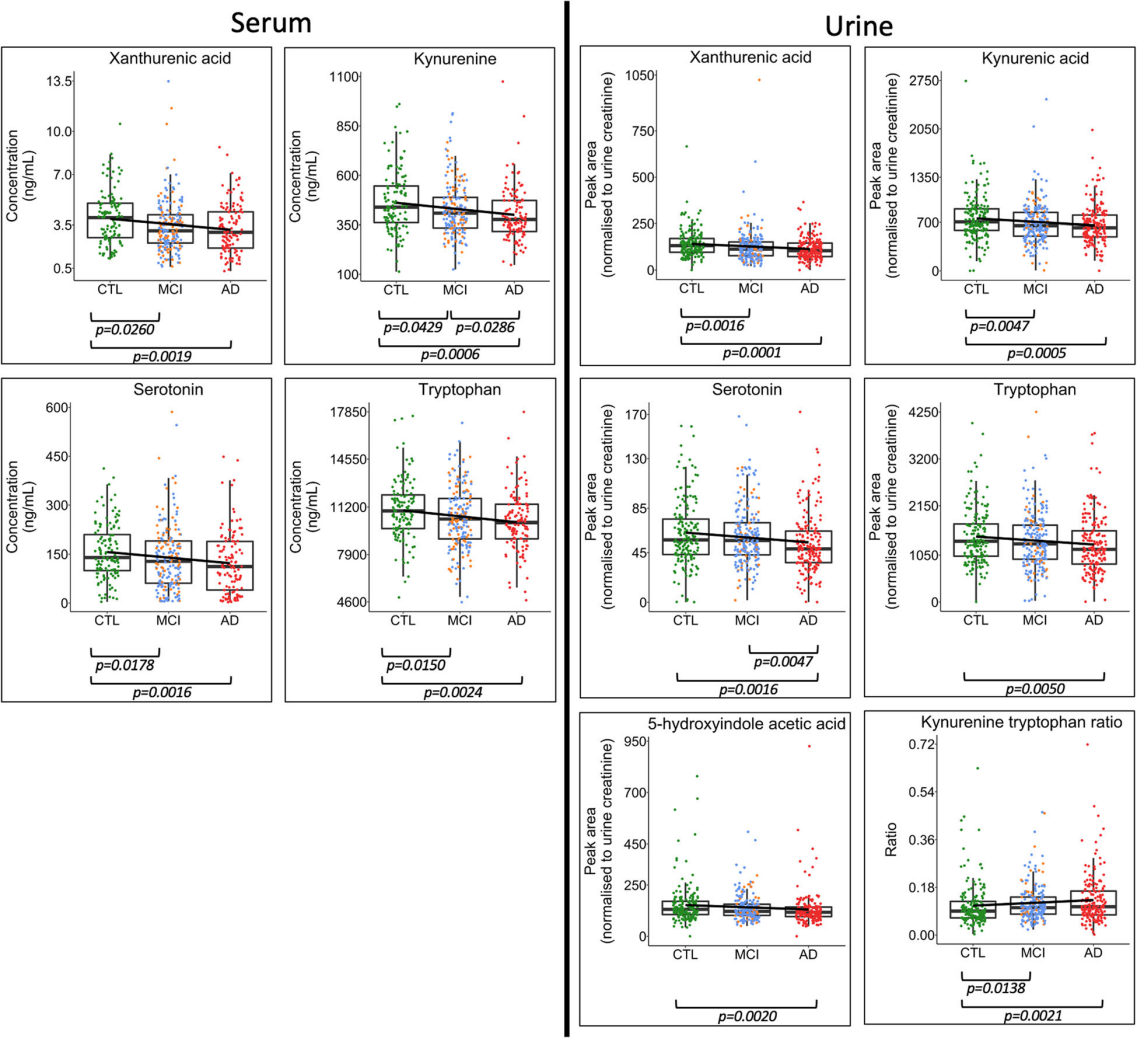
Inter-group metabolite differences. Boxplots highlighting differences between metabolite concentrations in serum and urine when comparing AD (red), MCI (blue = sMCI, yellow = cMCI) and age-matched controls (CTL—green). Boxplots are shown for metabolites in the serotonin and kynurenine pathways that reported significant differences following univariate Kruskal-Wallis tests. Figure *p* values were calculated using Dunn’s post hoc test for those metabolites that reported a Kruskal-Wallis *p* value < 0.1 following adjustment for multiple testing using the Holm method. A decreasing trend was observed: control > MCI > AD for each metabolite. This trend was observed regardless of gender (Fig. 3). Boxplots for all metabolites are presented in Fig. S1 and S2

**Fig. 3:**
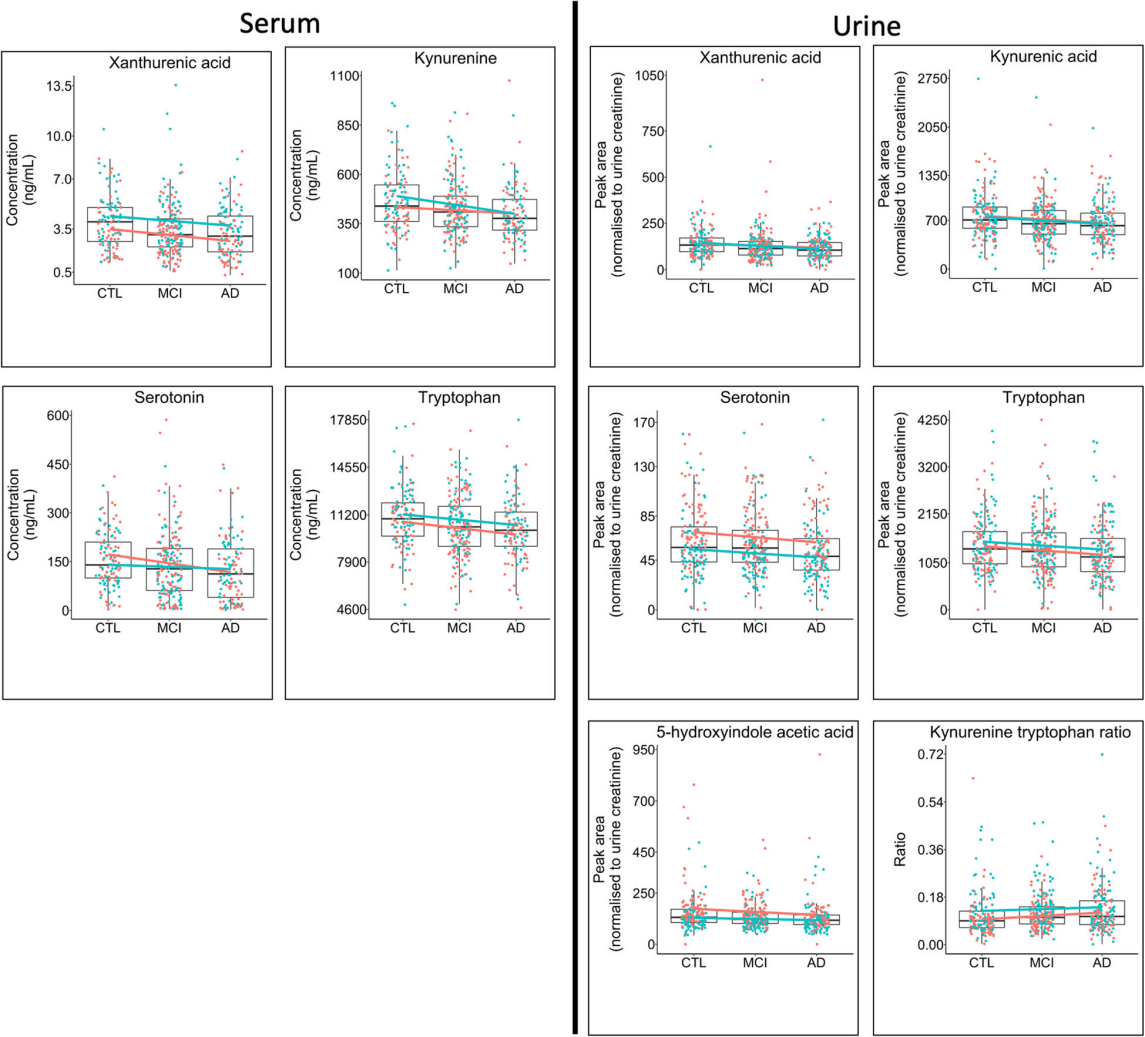
Inter-group metabolite differences stratified by gender. Boxplots highlighting differences between metabolite concentrations in serum when comparing Alzheimer’s disease (AD), mild cognitive impairment (MCI) and age-matched controls (CTL). Boxplots were fitted with a linear model coloured by gender (red = female, blue = male). Boxplots are shown for the metabolites in the serotonin and kynurenine pathways that reported significant differences between participant groups (Supplementary Figs. S1 and S2). The plots suggest that the observed lower concentrations in metabolites between groups CTL > MCI > AD follow a similar trend across both genders

An overall decreasing trend in metabolite cocentrations was also observed in the direction of control > MCI > AD for each metabolite identified as differentiating control samples from those from the cognitively impaired groups (Fig. 2). This trend was observed regardless of gender (Fig. 3).

### Study phase 2—Quantification of tryptophan pathway metabolites in serum

Data quality was assessed as described in supplementary methods, with all metabolite quantification calculated using a linear calibration dilution set (*r*^2^ > 0.990) and with analytical QCs calculated to be within 15% of the target concentration (20% for the LLOQ).

Kruskal-Wallis testing reported significant inter-group metabolite differences for tryptophan (*p* = 0.0047, Holm-adjusted *p* = 0.0756), kynurenine (*p* = 0.0019, Holm-adjusted *p* = 0.0340), xanthurenic acid (*p* = 0.0018, Holm-adjusted *p* = 0.0340) and serotonin (*p* = 0.0037, Holm-adjusted *p* = 0.0625) (Table 3).

For metabolites that reported a Holm-adjusted *p* value of < 0.1, post hoc Dunn’s tests were performed to investigate differences between the individual study groups. The key serum metabolites when comparing control to AD (Table 3) were xanthurenic acid (*p* = 0.0019), kynurenine (*p* = 0.0006), serotonin (*p* = 00016) and tryptophan (*p* = 0.0024). Serum metabolite concentrations were also reported to be significantly lower when comparing MCI to the control groups for xanthurenic acid (*p* = 0.0260), kynurenine (*p* = 0.0429), serotonin (*p* = 0.0178) and tryptophan (*p* = 0.0150) whilst kynurenine was observed to be significantly lower in AD compared to the MCI group (*p* = 0.0286).

An overall decreasing trend in serum metabolite concentrations was also observed: control > MCI > AD for each metabolite (Fig. 2). This trend was observed regardless of gender (Fig. 3).

### Study phase 3—Further investigation of key metabolites

#### Metabolite associations with participant age

Univariate Pearson correlation reported a negative association between serum tryptophan concentrations and an increase in participant age (*r* = − 0.1193, *p* = 0.0131, Holm-adjusted *p* = 0.0915). In contrast, a positive correlation was observed between serum kynurenine and participant age (*r* = 0.1858, *p* = 0.0001, Holm-adjusted *p* = 0.0010). No significant age-related associations were observed with the remaining serum metabolites (xanthurenic acid and serotonin) (Holm-adjusted *p* > 0.1).

**Fig. 4:**
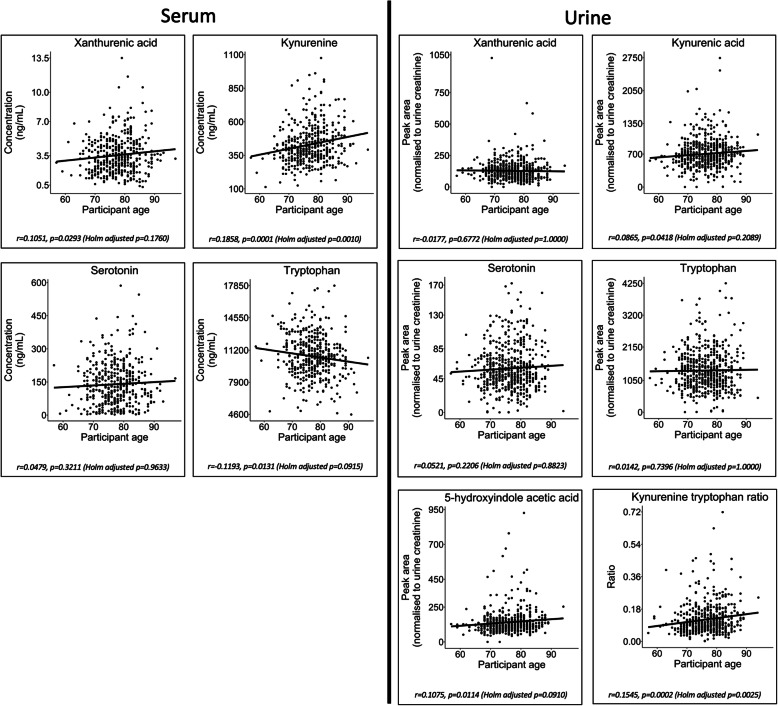
Metabolite associations with participant age. Plots presenting metabolite concentration change in association with participant age. The plots were fitted with a linear regression model. Plots are shown for the metabolites in the serotonin and kynurenine pathways that reported significant differences between participant groups (Supplementary Figs. S1 and S2). The plots suggest that only serum tryptophan has a negative correlation with age—a major risk factor of AD (*r* = − 0.1193). The remaining key metabolites all have positive correlation with increased age; however, only serum kynurenine and urine kynurenic acid have a significant positive correlation (serum kynurenine: *r* = 0.1858, *p* = 0.0001 (Holm-adjusted *p* = 0.0009) and kynurenic acid: *r* = 0.0865, *p* = 0.0418 (Holm-adjusted *p* = 0.2089))

A positive association was noted between urinary 5-hydroxyindoleacetic acid and participant age (*r* = 0.1075, *p* = 0.0114, Holm-adjusted *p* = 0.0910). The remaining four urine metabolites that were characteristically altered in AD (xanthurenic acid, kynurenic acid, serotonin and tryptophan) were not found to be significantly associated with age (Holm-adjusted *p* > 0.1) (Table S1 and Fig. 4).

#### Metabolite associations with participant MMSE scores

**Fig. 5:**
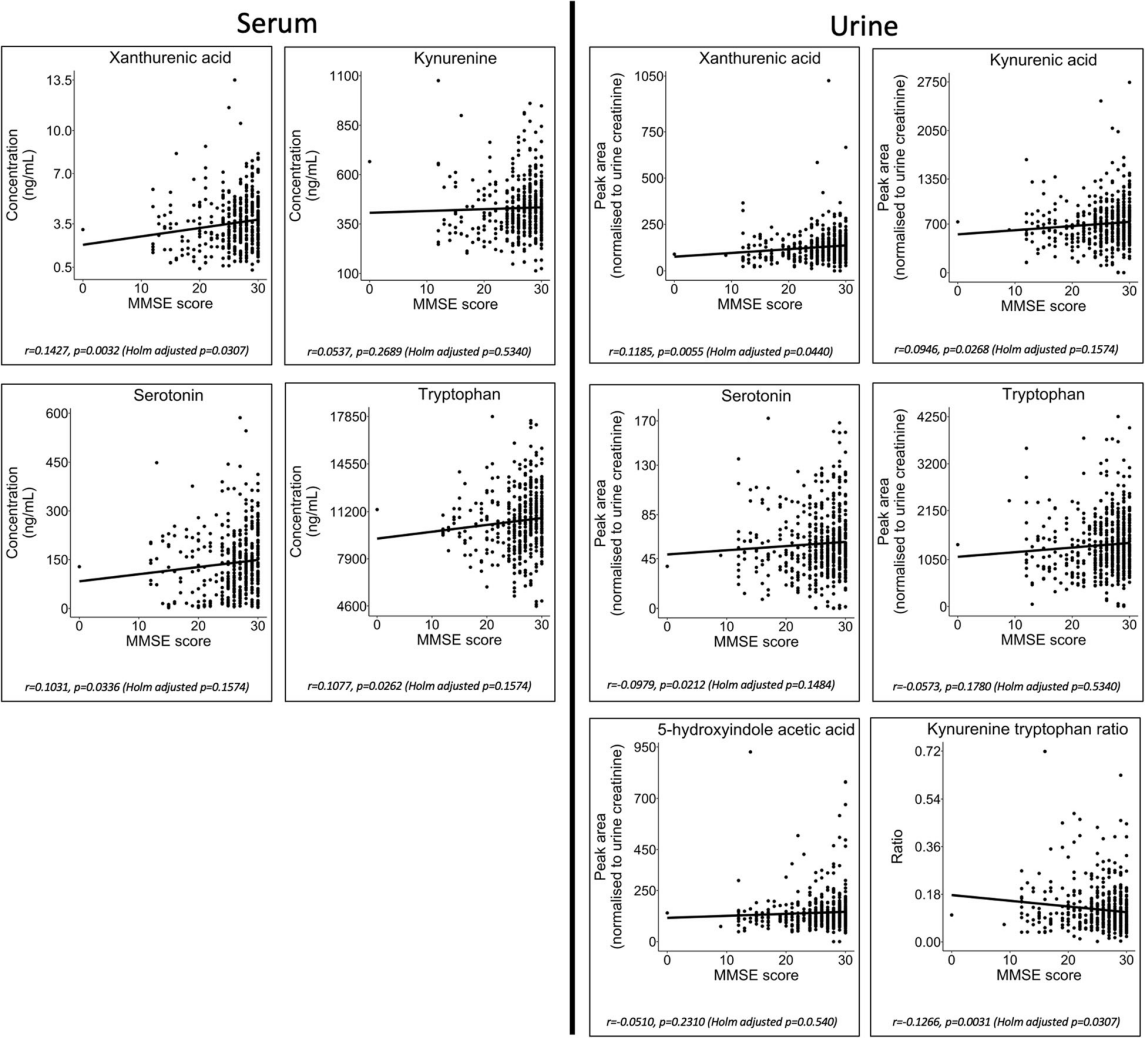
Serum and urine metabolite associations with participant MMSE scores. Plots presenting metabolite concentration change in association with participant Mini-Mental State Examination (MMSE) score. The plots were fitted with a linear regression model. Plots are shown for the metabolites in the serotonin and kynurenine pathways that reported significant differences between participant groups (Supplementary Figs. S1 and S2). The plots suggest that only urine xanthurenic acid has a significant negative correlation with participant MMSE (*r* = − 0.1266, *p* = 0.0031 (Holm-adjusted *p* = 0.0307)). A significant positive correlation was observed between MMSE score and both urine kynurenic acid (*r* = 0.1427, *p* = 0.0032 (Holm-adjusted *p* = 0.0307)) and serotonin (*r* = 0.1185, *p* = 0.0055 (Holm adjusted))

Univariate Pearson correlation reported a significant negative association between participant MMSE score and urine kynurenine/tryptophan ratio (*r* = − 0.1266, *p* = 0.0031, Holm-adjusted *p* = 0.0307). A significant positive correlation was observed between MMSE score and xanthurenic acid in both urine (*r* = 0.1427, *p* = 0.0032, Holm-adjusted *p* = 0.0307) and serum (*r* = 0.1185, *p* = 0.0055, Holm adjusted (Table S2 and Fig. 5)).

#### Metabolite comparison between MCI subgroups

**Fig. 6:**
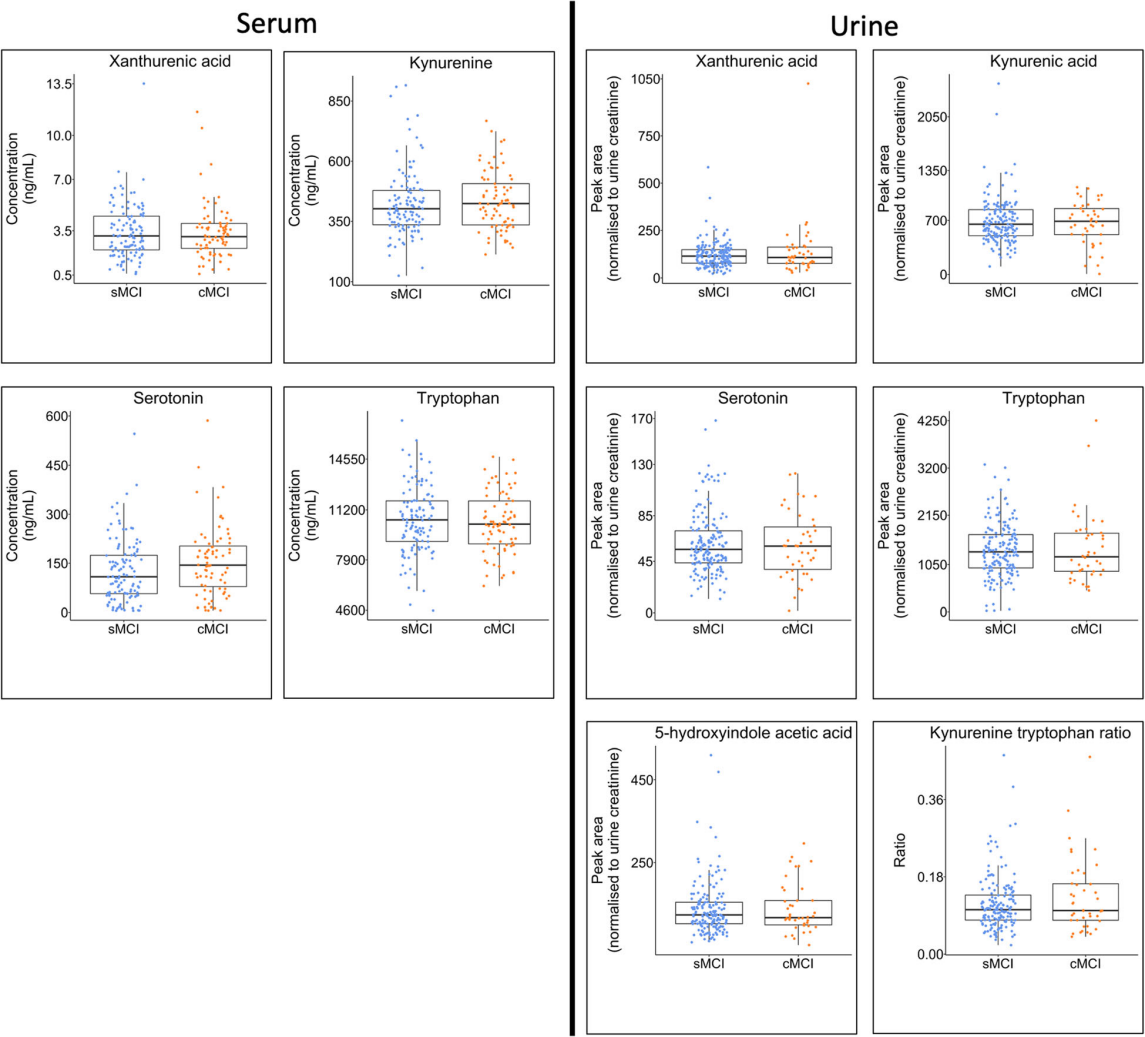
Metabolite comparison between mild cognitive impairment subgroups. Boxplots highlighting differences between metabolite concentrations in serum when comparing two subgroups of participants with a baseline clinical diagnosis of mild cognitive impairment (MCI). The first group (blue) remained cognitively stable throughout follow-up study visits (stable MCI (sMCI)), whilst the second group (yellow) experienced a deterioration in cognition and converted to a clinical diagnosis of AD at follow-up (converting MCI (cMCI)). Boxplots are only presented for the metabolites in the serotonin and kynurenine pathways that reported significant differences between control, MCI and AD participant groups in phases 1 and 2 of the study (Supplementary Figs. S1 and S2). No significant differences were observed between the sMCI and cMCI groups in this study

Univariate Mann-Whitney *U* tests reported no significant differences between participants diagnosed with MCI at the baseline visit who remained stable (sMCI) and those who converted to a diagnosis of AD at later follow-up visits (cMCI) (Table S3 and Fig. 6).

#### Metabolite associations across biofluids

**Fig. 7:**
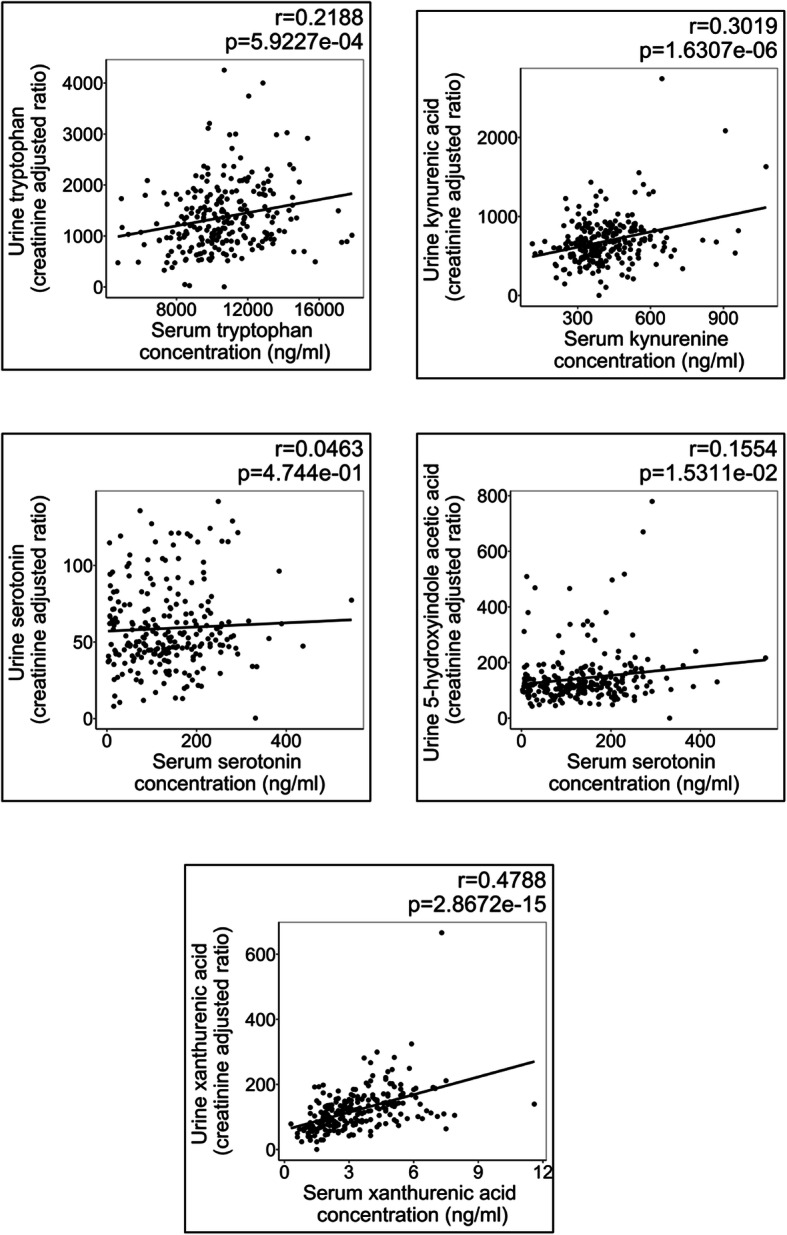
Metabolite associations across biofluids. Scatter plots fitted with a linear regression describing the correlation between significant serum and urine metabolites. Correlations were calculated where both biofluids from a single individual were available. Significant positive correlations were observed for serum tryptophan/urine tryptophan, serum kynurenine/urine kynurenic acid, serum xanthurenic acid/urine xanthurenic acid and serum serotonin/urine 5-indoleacetic acid; however, serum serotonin/urine serotonin did not demonstrate a significant correlation

Pearson correlation analysis comparing the metabolites across the two biofluids reported a positive correlation between urine and serum levels of tryptophan (*r* = 0.2188, *p =* 0.0006, Holm-adjusted *p =* 0.0018) and xanthurenic acid (*r* = 0.4788, *p* = 2.867e^−15^, Holm-adjusted *p* = 1.4336e^−14^). Kynurenine and serotonin did not correlate across the biofluids; however, serum concentrations were positively correlated with their respective polar urinary metabolites kynurenic acid and 5-hydroxyindoleacetic acid (serum kynurenine | urine kynurenic acid *r* = 0.3019, *p* = 1.631e^−6^, Holm-adjusted *p* = 6.5229e^−6^ and serum serotonin | urine 5-hydroxyindoleacetic acid *r* = 0.1554, *p* = 0.0153, Holm-adjusted *p* = 0.0306) (Table S4 and Fig. 7).

#### Impact of selective serotonin reuptake inhibitor (SSRI) medication

**Fig. 8:**
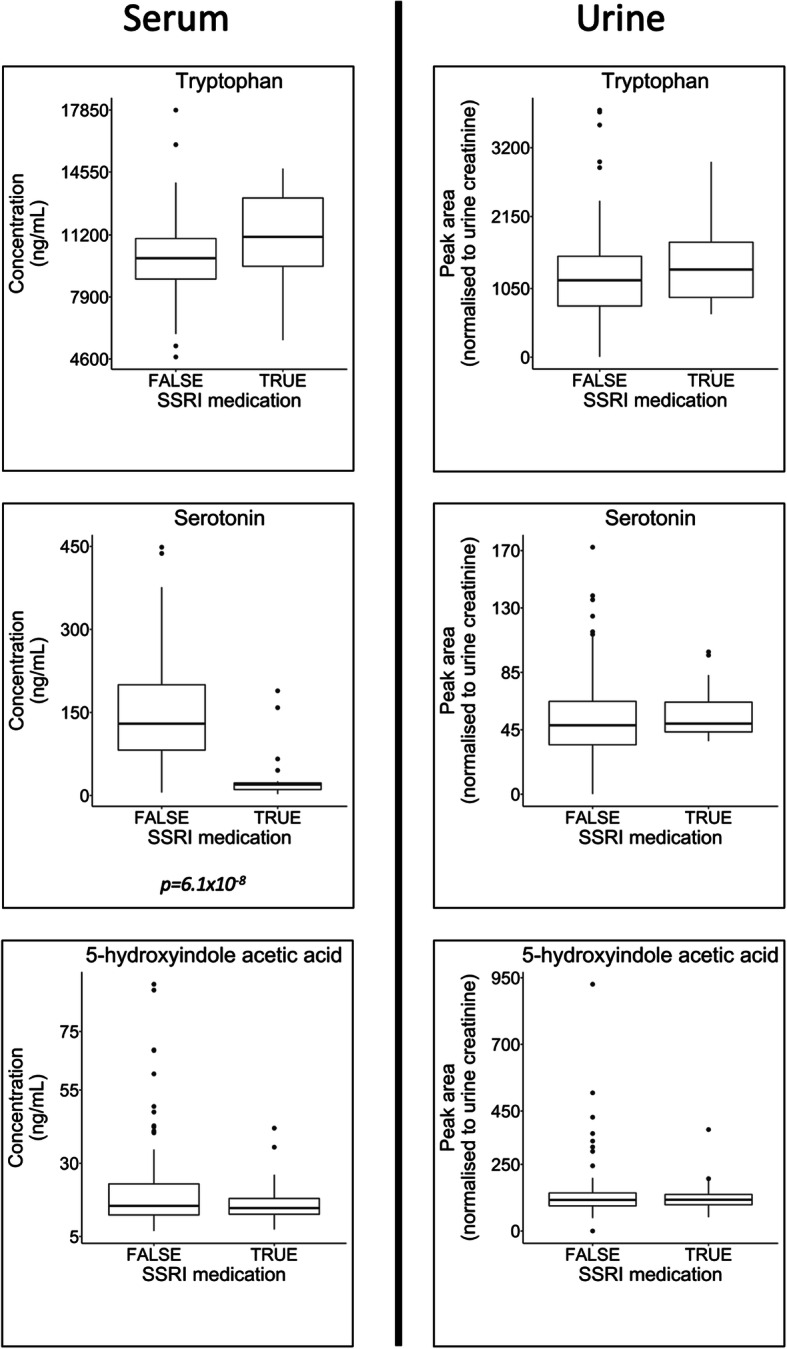
Impact of selective serotonin reuptake inhibitor (SSRI) medication. Boxplots highlighting differences between study participants from the AD study group who had been prescribed SSRI medication and those who had not. There were no significant differences (analysis by Mann-Whitney *U* tests) when comparing the metabolites tryptophan and the downstream metabolite 5-hydroxyindoleaceitic acid in both urine and serum. There was also no significant difference in serotonin in urine; however, in serum, there were significantly lower levels of serotonin in the AD group who were prescribed SSRI medication for depression. Further longitudinal work would be required to determine if this is a result of the SSRI medication or due to the underlying pathophysiology of the individual that leads to treatment for depressive symptoms

Study participants diagnosed with AD who were prescribed SSRI medication had significantly lower levels of serotonin than AD study participants with no reported SSRI intake (*p* = 6.1e^−8^, Holm-adjusted *p* = 1.10e^−6^) (Table S5 and Fig. 8). Kruskal-Wallis analysis of a sub-cohort of samples consisting only of participants with no reported SSRI medication is displayed in Table S6. In urine, xanthurenic acid (*p* = 0.001, Holm-adjusted *p* = 0.0013), kynurenic acid (*p* = 0.0006, Holm-adjusted *p* = 0.0044), serotonin (*p* = 0.0009, Holm-adjusted *p* = 0.0061), 5-hydroxyindoleacetic acid (*p* = 0.0070, Holm-adjusted *p* = 0.0309), tryptophan (*p* = 0.0051, Holm-adjusted *p* = 0.0309), 3-hydroxyanthranilic acid (*p* = 0.0083, Holm-adjusted *p* = 0.0309) and the kynurenine/tryptophan ratio (*p* = 0.0052, Holm-adjusted *p* = 0.0309) were significantly different between the groups. In serum, xanthurenic acid (*p* = 0.0027, Holm-adjusted *p* = 0.0440), kynurenine (*p* = 0.0006, Holm-adjusted *p* = 0.0099) and tryptophan (*p* = 0.0009, Holm-adjusted *p* = 0.0152) demonstrated significant differences between study groups; however, no significant difference was found in serotonin concentrations (*p* = 0.1286, Holm-adjusted *p* = 1.0000).

## Discussion

### The kynurenine pathway in Alzheimer’s disease

Significantly lower serum tryptophan, kynurenine and xanthurenic acid were found in participants clinically diagnosed with AD compared with controls, whilst serum xanthurenic acid demonstrated a significant positive correlation with participant MMSE cognitive scores.

Previous literature regarding tryptophan pathway metaboltes in AD contains conflicting results. Lower levels of tryptophan, xanthurenic acid, 3-hydroxyanthranilic acid [18] and tryptophan [33] and tryptophan and kynurenic acid [9] have been reported in the plasma of AD patients in agreement with our findings. However, conversely there are conflicting reports of higher levels of serum 3-hydroxykynurenine in AD [19], higher levels of serum kynurenine and anthranilic acid in females with a high neocortical amyloid-β load [10] and positive correlations between serum kynurenine metabolites with plasma amyloid-β_(1–42)_ and neurofilament light chain [11]. This reported discrepancy may be a consequence of the relatively small sample sizes typically used for analysis, emphasising the need for further investigation into the association between AD and tryptophan metabolism using larger cohorts.

In addition, the metabolites kynurenic acid and quinolinic acid have been reported to be significantly higher in the cerebrospinal fluid (CSF) of individuals clinically diagnosed with AD [33]. The same study reported no significant differences in plasma concentrations of these metabolites collected from the same study participants. This finding is in agreement with the serum data presented here, where we report no significant differences of the two metabolites when comparing between the three clinical groups. Our data did however demonstrate a trend that suggested that kynurenic acid was lower in the individuals diagnosed with AD. Although this finding was significant in the initial Kruskal-Wallis inter-group test, it did not pass the correction for multiple testing threshold for the study. This result may suggest that metabolic changes in the kynurenine pathway observed in CSF may differ to those seen in blood-based biofluids. A study consisting of 20 AD cases and 18 controls that analysed the kynurenine pathway in both plasma and CSF reported significant metabolite correlations across CSF and plasma for kynurenine, 3-hydroxykynurenine, anthranilic acid, picolinic acid and neopterin; however, kynurenic acid was not significantly correlated across the biofluids [12]. In future, a large cohort study with matched sample types including CSF, serum/plasma and urine collected at the same study visit would provide valuable information on the translation of metabolic biomarkers in AD across the different biofluid compartments.

Previous literature has reported associations between quinolinic acid and Alzheimer’s disease pathology [34, 35] with reports of β-amyloid inducing the production of quinolinic acid by macrophages and microglia in vitro [36]. However, our data reported no significant differences between the concentrations of quinolinic acid in control and AD participant groups (Table 3). Likewise, the concentrations of picolinic acid have also been previously reported to associate with AD pathology [12]. Again, our data did not show any significant differences in the serum concentrations of picolinic acid between the participant groups (Table 3). The reasons behind the disparity in previous literature reports and our data are unclear but may be because the previous literature has typically compared the metabolites with specific features of AD pathology rather than overall clinical classification. Future large-scale studies that are able to collect pathological data in tandem with the clinical, cognitive and metabolic data may reveal more valuable information about these apparent relationships.

In urine, we also found significantly lower levels of tryptophan, xanthurenic acid and kynurenic acid, and urine xanthurenic acid demonstrated a significant positive correlation with participant MMSE cognitive scores. To the best of the authors’ knowledge, comparisons of urinary kynurenines in clinical cases of AD, MCI and controls have not been previously reported. The lower levels observed of the three urinary metabolites are consistent with our findings in serum.

Mechanistically, the rate limiting enzyme in the kynurenine metabolic pathway is indoleamine 2,3-dioxygenase (IDO)—a critical enzyme in systemic inflammation expressed by key cells of the immune system, including microglia [37]. The activity of IDO can be monitored using the circulating kynurenine/tryptophan ratio [16]. Here, we showed a higher urinary kynurenine/tryptophan ratio in the AD group and a significant negative correlation of the ratio with participant MMSE score, suggesting increased conversion of tryptophan to kynurenine prior to renal excretion, perhaps as a result of systemic inflammation and IDO upregulation in cases of AD and cognitive decline [38].

Our data also found no significant differences when comparing metabolite concentrations between participant groups for NAD^+^ and its precursors nicotinic acid, nicotinic riboside and β-nicotinamide mononucleotide. NAD is a key functional metabolite in cellular metabolism and has been hypothesised as playing a role in the disrupted energy metabolism pathways that occur in AD [39]. To the authors’ knowledge, there are no literature references that directly compare differences in the concentration of blood- or urine-based nicotinamide pathway metabolites in AD. However, there have been many examples of in vivo murine model work that has investigated the potential use of nicotinic metabolites as a treatment to slow AD pathology [40–42]. Our data suggest that these metabolites are not present at different concentrations in circulatory serum when comparing between the participant groups; however, further investigation into alternative biofluids such as CSF or post-mortem brain would add useful information regarding the role of the nicotinic pathway in AD.

### The serotonin pathway in Alzheimer’s disease

A consequence of lower tryptophan bioavailability and an increase IDO enzyme activity is a reduced capacity for serotonin biosynthesis. This is reflected in our results, with lower levels of serotonin and 5-hydroxyindole acetic acid reported in the AD group.

Despite reports of lower amounts of serotonin in cerebrospinal fluid [43] and post-mortem brains [44, 45] in AD, to the best of the authors’ knowledge, differences in blood or urine have not been previously published and are reported here for the first time.

However, serotonin and serotonergic signalling have previously been proposed to be disrupted in AD [3], including reports of an increase in serotonin-4 receptors in the brain in response to an increased amyloid burden [46]. Madsen et al. hypothesised that this may be a consequence of lower serotonin, thereby acting as a compensatory effect to improve cognitive function, to increase acetylcholine release or to counteract increased amyloid accumulation [46]. Subsequent studies in mice have reported that amyloid precursor protein processing is regulated by the serotonin-4 receptor and activation of serotonin-4 receptor upregulates α-secretase, resulting in the formation of soluble amyloid, rather than the insoluble amyloid-β otherwise produced by cleavage via the β- and γ-secretase route [5]. Receptor agonists of serotonin-4 receptor, serotonin-5 receptor and serotonin-6 receptor have also been shown to reduce brain interstitial fluid levels of amyloid-β in the brains of mouse models [47].

In addition, selective serotonin reuptake inhibitors (SSRIs) are under investigation as therapeutic agents in AD. SSRIs work by increasing free serotonin at the synapse or neuronal cells resulting in increased levels of free serotonin available to synaptic receptors [14]. In both mouse models and humans, SSRIs have been reported to reduce levels of interstitial brain amyloid-β [48].

SSRIs are currently licenced for use in depression, and therefore, the study contained samples collected from participants who reported prescription of SSRI medication across all of the participant groups (Table 1). The mechanism of SSRI action is not fully understood, and there are conflicting reports in the literature regarding the effect of SSRI medication on blood and urine levels of serotonin [49–51]. In our data, individuals who were prescribed SSRI medication had significantly lower levels of serotonin in their serum; however, this difference was not observed in urine (Fig. 7). Further longitudinal work would be required to interpret whether this observation is a direct result of the SSRI medication, or due to the underlying pathophysiology of the individual that leads to treatment for depressive symptoms, a condition frequently linked to lower levels of plasma and serum serotonin [49].

To assess the impact of SSRI on the overall result of the study, univariate analysis was repeated only with the participants who did not take SSRI medication. In the re-analysis, serum serotonin no longer reported significant differences between AD and control groups (Table S5). However, interestingly, results in urine mirrored the full cohort, including the continued observation of significantly lower levels in the AD group of urinary tryptophan, serotonin and 5-hydroxyindoleacetic acid suggesting altered tryptophan and serotonin metabolism and renal excretion in the AD group, regardless of SSRI intake status. The discrepancy between serum and urine is unexplained, nevertheless the results raise important questions about the serotonergic signalling system in AD. Future longitudinal phenotyping resulting in accurate patient stratification may enable greater insight into the impact of serotonin bioavailability and SSRI medication in AD.

### The bioavailability of tryptophan in Alzheimer’s disease

**Fig. 9:**
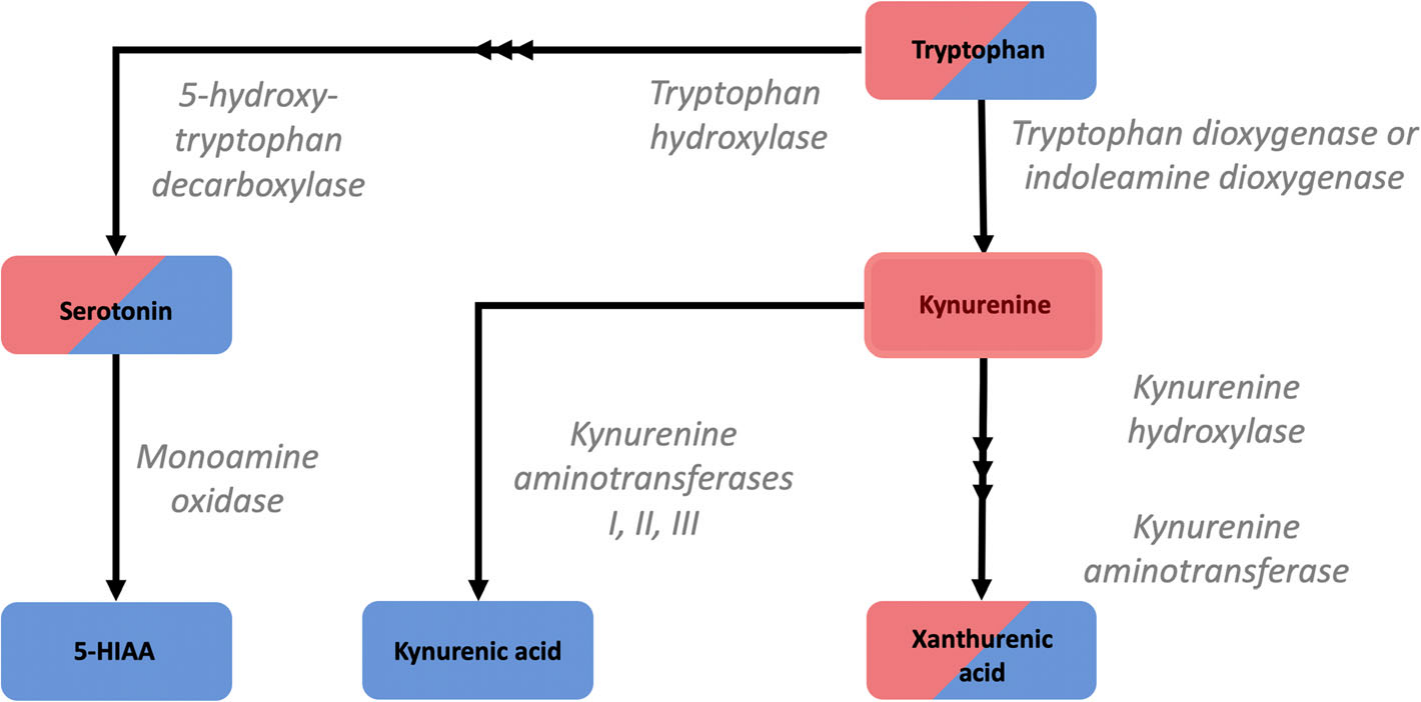
Tryptophan pathway. Pathway map presenting the key metabolites that reported significant inter-group differences following Kruskal-Wallis tests. Post hoc Dunn tests revealed that those highlighted with red shading were significantly lower in the AD group in serum, whilst those highlighted with blue shading were significantly lower in AD in urine. The downstream metabolites are inherently more polar and are therefore fit with biological and metabolic logic that polar, downstream metabolites would be renally excreted and therefore altered in urine

Tryptophan is the parent metabolite in both the serotonin and kynurenine pathways, and therefore, its bioavailability may have a downstream effect on the resultant bioavailability of key neuroactive metabolites in the circulatory system (Fig. 9).

As tryptophan is an essential amino acid that cannot be synthesised in mammalian systems, the bioavailability of circulatory free tryptophan is primarily influenced by the consumption of protein in the diet combined with the rate of usage in protein synthesis and the ability to absorb amino acids through the intestinal wall. In AD, the impact on bioavailability of essential amino acids is highly complex and multifactorial. Changes in appetite are well documented in AD with many occurrences of eating disturbances reported varying between both the loss and increase of appetite, as well as changes in dietary preference [52]. In addition, faecal calprotectin (a marker of intestinal inflammation) has been reported to be negatively associated with serum essential amino acids in individuals with AD—suggesting a disturbed intestinal barrier function leading to the lowering of essential amino acid blood concentrations [53].

The bioavailability of tryptophan is also known to be controlled by the population and diversity of an individual’s gut microbiome [54], with manipulation of the microbial composition demonstrated to impact plasma concentrations of tryptophan [55].

Research investigating alterations in the composition of the gut microbiome of individuals with AD have suggested that they have differences in the prevalence of Firmicutes, Bifidobacteria and Bacteroidetes compared with controls [56], all of which have been reported to possess tryptophan decarboxylase enzymes [54, 57], suggesting that gut diversity could impact the bioavailability of tryptophan and its downstream metabolites. However, in our results, the metabolite indole-3-acetic acid, an indole molecule known to be produced from tryptophan by gut bacteria [54], remained unchanged between participant groups (Kruskal-Wallis *p* = 0.8518, Holm-adjusted *p* = 1.0000), suggesting that the differences observed here in tryptophan metabolite may not be attributable to alterations in the gut microciome. Future studies warrant further investigation of indole containing metabolites (e.g. indole, tryptamine, indole lactic acid, indole aldehyde and indole propionic acid), which when combined with microbiome sequencing in a sample from AD cohorts will establish if any associations exist between tryptophan bioavailability, indole metabolites and gut microbial diversity.

## Conclusions

Here, we report significantly lower concentrations of tryptophan with downstream ramifications for the kynurenine and serotonin pathways in individuals clinically diagnosed with AD. Lower concentrations of metabolites involved in tryptophan metabolism were observed in both the urine and serum of participants and, in general, showed a declining trend for MCI. The results reported are based on the analysis of the largest cohort study to date that has investigated tryptophan metabolism in AD. Furthermore, the tryptophan-serotonin pathway may represent an easily modifiable pathway for influencing and managing the progression of AD and alleviating serotonergic signalling disruption in AD.

Future studies that are designed to investigate associations of tryptophan metabolism pathways with additional pathological markers of AD and cognitive decline, that were unavailable for the samples used in this study (e.g. cerebral amyloid load and/or emerging circulatory blood biomarkers such as p-tau 181 [58]), would provide valuable insight and further understanding of the metabolic processes that result in the kynurenine and serotonin pathway perturbations in AD.

## Supplementary Information

**Additional file 1.** All data de-identified (CSV 186 kb)

**Additional file 2.** Supplementary methods, tables and figures

## Data Availability

All data generated or analysed during this study are included in this published article [and its supplementary information files].

## Abbreviations

AD: Alzheimer’s disease
IDO: Indoleamine 2,3-dioxygenase
MCI: Mild cognitive impairment
SSRI: Selective serotonin reuptake inhibitor
TDO: Tryptophan 2,3-dioxygenase
DCR: Dementia Case Register
sMCI: Stable MCI
cMCI: Converting MCI
UHPLC-QTOF-MS: Ultra-high-performance liquid chromatography quadrupole-time-of-flight mass spectrometry
^1^H-NMR: Proton nuclear magnetic resonance
UHPLC-MS/MS: Ultra-high-performance liquid chromatography tandem mass spectrometry
