# Supplementary material for: Metabolic phenotyping reveals a reduction in the bioavailability of serotonin and kynurenine pathway metabolites in both the urine and serum of individuals living with Alzheimer’s disease
Source: Alzheimers Res Ther. 2021 Jan 9;13:20. doi: 10.1186/s13195-020-00741-z (PMC7797094; doi:10.1186/s13195-020-00741-z)
Supplement: Supplementary file 2 — Additional file 2. Supplementary methods, tables and figures [file 13195_2020_741_MOESM2_ESM.docx]

**Metabolic phenotyping reveals a reduction in the bioavailability of serotonin and kynurenine pathway metabolites in both the urine and serum of individuals living with Alzheimer’s disease**

Luke Whiley^1,14,15^, Katie E. Chappell^2,3^, Ellie D’Hondt^4^, Matthew R. Lewis^2,3^, Beatriz Jiménez^3^, Stuart G. Snowden^5,+^, Hilkka Soininen^6,16^, Iwona Kłoszewska^7,16^, Patrizia Mecocci^8,16^, Magda Tsolaki^9,16^, Bruno Vellas^10,16^, Jonathan R. Swann^2^, Abdul Hye^10^, Simon Lovestone^11,12,16^, Cristina Legido-Quigley^5,13^, Elaine Holmes* ^1,2,14,15^

* Corresponding author

^1^ UK Dementia Research Institute, Imperial College London, Hammersmith Hospital, London, W12 0NN, United Kingdom

^2^ Section of Nutrition, Imperial College London, South Kensington Campus, London SW7 2AZ, United Kingdom

^3^ MRC-NIHR National Phenome Centre, Imperial College London, Hammersmith Hospital, London W12 0NN, United Kingdom

^4^ imec, Exascience Life Lab, Kapeldreef 75, B-3001 Leuvne, Belgium

^5^ King’s College London, Institute of Psychiatry, Psychology and Neuroscience, London, UK

^6^ Department of Neurology, University of Eastern Finland and Kuopio University Hospital, Kuopio, Finland.

^7^ Medical University of Lodz, Lodz, Poland.

^8^ Institute of Gerontology and Geriatrics, University of Perugia, Perugia, Italy.

^9^ 3rd Department of Neurology, Aristotle University, Thessaloniki, Greece.

^10^ INSERM U 558, University of Toulouse, Toulouse, France

^11^ Department of Psychiatry, Warneford Hospital, University of Oxford, Oxford, UK

^12^ Current affiliation at Janssen-Cilag Ltd

^13^ Steno Diabetes Center Copenhagen, Gentofte, Denmark

^14^ Health Futures Institute, Murdoch University, Perth WA 6105, Australia

^15^ The Perron Institute for Neurological and Translational Science, Nedlands, WA 6009, Australia

^16^ on behalf of AddNeuroMed consortium

^+^ Present address - Core Metabolomics and Lipidomics Laboratory, Metabolic Research Laboratories, Institute of Metabolic Science, University of Cambridge, CB2 0QQ, UK.

**Keywords:** Alzheimer’s Disease/kynurenine/tryptophan/serotonin/metabolic phenotyping/mass spectrometry/systemic inflammation/serotonergic signalling

**Supplementary methods**

**Study phase 1 – Metabolite profiling of urine**

**Urine UHPLC-QTOF-MS metabolic profiling data acquisition**

Urine samples were randomized and transferred to 96 well plates. Samples were then diluted 1:1 in water before 2 µL was injected on the UHPLC-MS instrumentation.

Untargeted metabolite profiling data was acquired using a Waters Acquity UHPLC system coupled to a Waters Xevo G2 quadrupole-time-of-flight mass spectrometer (QTOF-MS). Chromatographic separation of metabolites was completed on a reversed phase Waters Acquity HSS T3 C_18_ column (150mm x 2.1mm x 1.8µm) using a 15-minute gradient method. QTOF-MS data were acquired in positive ionization mode, and m/z data were collected between 50-1200 Da.

Two sets of quality control (QC) samples were used - the first, termed long-term reference (LTR), was a urine pool collected from donors external to the study and was used as an external system performance benchmark. The second QC set, termed the study reference (SR), was a urine pool prepared with equal volumes of each study urine sample and was therefore representative of the study population, which was used to assess intra-study data quality and instrument performance.

**Urine metabolite feature selection and data processing**

Eight key metabolites of tryptophan metabolism were measured (**Table 3**). Metabolites were annotated by matching the retention times and mass-to-charge-ratios of metabolic features in the MS profiles of urine to those obtained from the MS analysis of commercial analytical standards. Peak integrals were extracted using Skyline 4.2 (MacCross Lab Software) ^37^. Peak integrals were then normalized using creatinine quantification values derived from ^1^H-NMR spectral data.

**Proton Nuclear Magnetic Resonance (^1^H-NMR) parameters for the quantification of creatinine in urine**

540 µL of urine was transferred to an NMR tube (4” length x 5mm diameter) and spiked with 60 µL of potassium phosphate buffer (1.5 M at pH 7.4) containing 3-(Trimethyl-silyl) propionic acid-d4 sodium salt (TSP, 5.8 mM) and sodium azide (NaN_3_, 2 mM). This was performed using a Gilson 215 liquid handler robot. ^1^H NMR analysis was performed on a 600 MHz Avance III HD Bruker spectrometer at 300 Kelvin using a 4 minutes acquisition method consisting of 32 scans. The relaxation delay was 4 s, spectral windows was 20 ppm divided into 65000 data points. Experiments were acquired using TopSpin 3.2 and ICON NMR. Phasing, baseline correction and calibration to TSP were carried out in automation after each acquisition.

**Study phase 2 – Quantification of metabolites in serum**

**Serum UHPLC-MS/MS quantification of tryptophan metabolites**

Serum samples were randomised and transferred to 96 well plate format. 30 µL of serum was spiked with SIL internal standards. Protein precipitation and removal was performed using a two-step protocol consisting of addition of methanol followed by pass-through solid phase extraction. Samples were then taken to dryness under nitrogen and re-suspended in 10 mM ammonium formate with 0.5 % formic acid. 5 µL was then injected onto the UHPLC-MS/MS system.

Data acquisition was performed on a Waters Acquity UHPLC system coupled to a Waters TQ-S tandem mass spectrometer (UHPLC-MS/MS). Chromatographic separation was performed on a reversed phase Waters Acquity HSS T3 C_18_ column (150mm x 2.1mm x 1.8µm) using an optimised 7 -minute solvent gradient. MS selective reaction monitoring (SRM) transitions were optimised for each metabolite to ensure an appropriate sensitivity and linear range for metabolites in a typical physiological serum sample.

Two sets of quality control (QC) samples were used - the first, termed the analytical QCs, were prepared using a range of known concentrations of analytical standards. The analytical QCs underwent the same extraction as samples and were used to assess the precision and accuracy of metabolite quantification across the plate in relation to the calibration curve. The second QC was termed the biological QCs, and were a pool of serum prepared with equal volumes of each study serum sample and was therefore representative of the study. The biological QC was used to observe the precision and accuracy of a repeat measurement in matrix across multiple plates of the project.

Raw data were processed in TargetLynx software package in MassLynx v4.2 (Waters). Data quality was assessed and accepted based upon US Food and Drug Administration (FDA) bioanalytical recommendations (<https://www.fda.gov/files/drugs/published/Bioanalytical-Method-Validation-Guidance-for-Industry.pdf>). To summarise, analytical quantification calibrations had to have a linearity of >0.990, with the calculated concentration of each individual standard within 15% of the theoretical prepared concentration (20% for the lowest limit of quantification [LLOQ]). QC samples prepared at known concentrations also had to return a calculated concentration within 15% (20% at LLOQ).

**Supplementary Tables**

**Table S1.** Results of Pearson correlation between metabolite concentrations and age. Corresponding scatterplots are presented in (**Figure** **4**)

| **Metabolite** | **Pearson correlation coefficient (r)** | ***p*** | **Holm adjusted *p*** |
| --- | --- | --- | --- |
| **urine - xanthurenic acid** | -0.0177 | 0.6772 | 1.0000 |
| **urine - kynurenic acid** | 0.0865 | 0.0418 | 0.2089 |
| **urine - serotonin** | 0.0521 | 0.2206 | 0.8823 |
| **urine - 5-hydroxyindoleacetic acid** | 0.1075 | 0.0114 | 0.0910 |
| **urine - tryptophan** | 0.0142 | 0.7396 | 1.0000 |
| **urine – kynurenine/tryptophan ratio** | 0.1545 | 0.0002 | 0.0025 |
| **serum - xanthurenic acid** | 0.1051 | 0.0293 | 0.1760 |
| **serum - kynurenine** | 0.1858 | 0.0001 | 0.0010 |
| **serum - serotonin** | 0.0479 | 0.3211 | 0.9633 |
| **serum - tryptophan** | -0.1193 | 0.0131 | 0.0915 |

**Table S2.** Results of Pearson correlation between metabolite concentrations and age. Corresponding scatterplots are presented in (**Figure** **5**)

| **Metabolite** | **Pearson correlation coefficient (r)** | ***p*** | **Holm adjusted *p*** |
| --- | --- | --- | --- |
| **urine - xanthurenic acid** | 0.1185 | 0.0055 | 0.0440 |
| **urine - kynurenic acid** | 0.0946 | 0.0268 | 0.1574 |
| **urine - serotonin** | 0.1031 | 0.0336 | 0.1574 |
| **urine - 5-hydroxyindoleacetic acid** | -0.0510 | 0.2310 | 0.5340 |
| **urine - tryptophan** | -0.0573 | 0.1780 | 0.5340 |
| **urine – kynurenine/tryptophan ratio** | -0.1266 | 0.0031 | 0.0307 |
| **serum - xanthurenic acid** | 0.1427 | 0.0032 | 0.0307 |
| **serum - kynurenine** | 0.0537 | 0.2689 | 0.5340 |
| **serum - serotonin** | 0.1031 | 0.0336 | 0.1574 |
| **serum - tryptophan** | -0.0573 | 0.1780 | 0.5340 |

**Table S3.** Results of univariate Mann Whitney U tests comparing study participants diagnosed with MCI at baseline visit who remained stable throughout follow-up visits (sMCI) or cognitively declined resulting in a conversion to a diagnosis of AD (cMCI). Corresponding boxplots are presented in (**Figure** **6**).

| **Metabolite** | ***p*** | **Holm adjusted *p*** |
| --- | --- | --- |
| **urine - xanthurenic acid** | 0.9761 | 1.0000 |
| **urine - kynurenic acid** | 0.8338 | 1.0000 |
| **urine - serotonin** | 0.8944 | 1.0000 |
| **urine - 5-hydroxyindoleacetic acid** | 0.9806 | 1.0000 |
| **urine - tryptophan** | 0.6306 | 1.0000 |
| **urine – kynurenine/tryptophan ratio** | 0.5985 | 1.0000 |
| **serum - xanthurenic acid** | 0.7528 | 1.0000 |
| **serum - kynurenine** | 0.3636 | 1.0000 |
| **serum - serotonin** | 0.0321 | 0.2886 |
| **serum - tryptophan** | 0.5671 | 1.0000 |

**Table S4 –** Results of Pearson correlation analysis of key metabolites across both serum and urine biofluids. Correlations were calculated where both biofluids from a single individual were available. Significant positive correlations were observed for: serum tryptophan/urine tryptophan; serum kynurenine/urine kynurenic acid; serum xanthurenic acid/urine xanthurenic acid; serum serotonin/urine 5-indoleacetic acid, suggesting a direct relationship between the levels of circulating metabolites in serum and those excreted in urine. However, serum serotonin/urine serotonin did not demonstrate significant correlation. Scatter plots are presented in **Figure 7.**

| **Urine metabolite** | **Serum metabolite** | **Pearson correlation coefficient (r)** | ***p*** | **Holm adjusted *p*** |
| --- | --- | --- | --- | --- |
| kynurenic acid | kynurenine | 0.3019 | 1.631e^-6^ | 6.5229e^-6^ |
| xanthurenic acid | xanthurenic acid | 0.4788 | 2.867e^-15^ | 1.4336e^-14^ |
| tryptophan | tryptophan | 0.2188 | 0.0006 | 0.0018 |
| serotonin | serotonin | 0.0463 | 0.4744 | 0.4744 |
| 5-hydroxyindole acetic acid | serotonin | 0.1554 | 0.0153 | 0.0306 |

**Table S5 –** Results of univariate Mann Whitney U tests comparing study participants diagnosed with AD at baseline who were prescribed SSRI medication, compared with participants diagnosed with AD at baseline who were not reported to be taking any SSRI medication. Data is presented in the manuscript in **Figure 8.**

| **Metabolite** | ***p*** | **Holm adjusted *p*** |
| --- | --- | --- |
| **urine - xanthurenic acid** | 0.3953 | 1.0000 |
| **urine - kynurenic acid** | 0.8477 | 1.0000 |
| **urine - serotonin** | 0.1583 | 1.0000 |
| **urine - 5-hydroxyindoleacetic acid** | 0.9560 | 1.0000 |
| **urine - tryptophan** | 0.2410 | 1.0000 |
| **urine – kynurenine/tryptophan ratio** | 0.6100 | 1.0000 |
| **serum - xanthurenic acid** | 0.8146 | 1.0000 |
| **serum - kynurenine** | 0.2219 | 1.0000 |
| **serum - serotonin** | 6.1080^-08^ | 1.0994e^-06^ |
| **serum - tryptophan** | 0.0220 | 0.3739 |

**Table S6 -** Summary of Kruskal-Wallis univariate analysis of participants with no reported SSRI intake. p values from Kruskal Wallis and subsequent post-hoc tests using Holm test to correct for multiple testing. Metabolites with a Holm adjusted p value of <0.1 are highlighted in bold and underwent a post-hoc Dunn’s test to observe differences between sub-groups. Metabolites with a Holm adjusted p value of > 0.1 did not undergo a post-hoc Dunn’s test and are labelled accordingly with NA.

| **Metabolite** | ***p* value (Kruskal-Wallis test)** | **adjusted *p* value (Holm)** | **Dunn’s post-hoc test *p* value** | | |
| --- | --- | --- | --- | --- | --- |
|  |  |  | **CTL - AD** | **CTL - MCI** | **MCI - AD** |
| **urine - xanthurenic acid** | 0.0001 | 0.0013 | 0.0022 | 0.0001 | 0.1206 |
| **urine - kynurenic acid** | 0.0006 | 0.0044 | 0.0018 | 0.0006 | 0.2636 |
| **urine - serotonin** | 0.0009 | 0.0061 | 0.1735 | 0.0005 | 0.0049 |
| **urine - 5-hydroxyindoleacetic acid** | 0.0070 | 0.0309 | 0.0722 | 0.0025 | 0.0681 |
| **urine - tryptophan** | 0.0051 | 0.0309 | 0.0651 | 0.0018 | 0.0607 |
| **urine – 3-hydroxyanthranilic acid** | 0.0083 | 0.0309 | 0.1009 | 0.0031 | 0.0528 |
| urine - 3-hydroxykynurenine | 0.4311 | 0.8622 | *NA* | *NA* | *NA* |
| urine - kynurenine | 0.9288 | 0.9288 | *NA* | *NA* | *NA* |
| **urine –kynurenine/tryptophan ratio** | 0.0052 | 0.0309 | 0.0111 | 0.0037 | 0.2577 |
|  | | | | | |
| **serum - xanthurenic acid** | 0.0027 | 0.0440 | 0.0041 | 0.0030 | 0.2543 |
| **serum - kynurenine** | 0.0006 | 0.0099 | 0.0222 | 0.0002 | 0.0209 |
| serum - serotonin | 0.1286 | 1.0000 | *NA* | *NA* | *NA* |
| **serum - tryptophan** | 0.0009 | 0.0152 | 0.0173 | 0.0003 | 0.0370 |
| serum - 3-hydroxyanthranilic acid | 0.0230 | 0.2765 | *NA* | *NA* | *NA* |
| serum - kynurenic acid | 0.0164 | 0.2299 | *NA* | *NA* | *NA* |
| serum - 3-hydroxykynurenine | 0.0207 | 0.2686 | *NA* | *NA* | *NA* |
| Serum - β-nicotinamide mononucleotide | 0.0098 | 0.1475 | *NA* | *NA* | *NA* |
| serum – picolinic acid | 0.1362 | 1.0000 | *NA* | *NA* | *NA* |
| serum - 5-hydroxyindoleacetic acid | 0.1308 | 1.0000 | *NA* | *NA* | *NA* |
| serum – nicotinic acid | 0.0788 | 0.8668 | *NA* | *NA* | *NA* |
| serum - quinolinic acid | 0.1658 | 1.0000 | *NA* | *NA* | *NA* |
| serum - dopamine | 0.5325 | 1.0000 | *NA* | *NA* | *NA* |
| serum - neopterin | 0.6160 | 1.0000 | *NA* | *NA* | *NA* |
| serum - nicotinic riboside | 0.7676 | 1.0000 | *NA* | *NA* | *NA* |
| serum citrulline | 0.7637 | 1.0000 | *NA* | *NA* | *NA* |
| serum - indole-3-acetic-acid | 0.9485 | 1.0000 | *NA* | *NA* | *NA* |
| serum - kynurenine/tryptophan ratio | 0.3344 | 1.0000 | *NA* | *NA* | *NA* |

**Supplementary Figures**

**
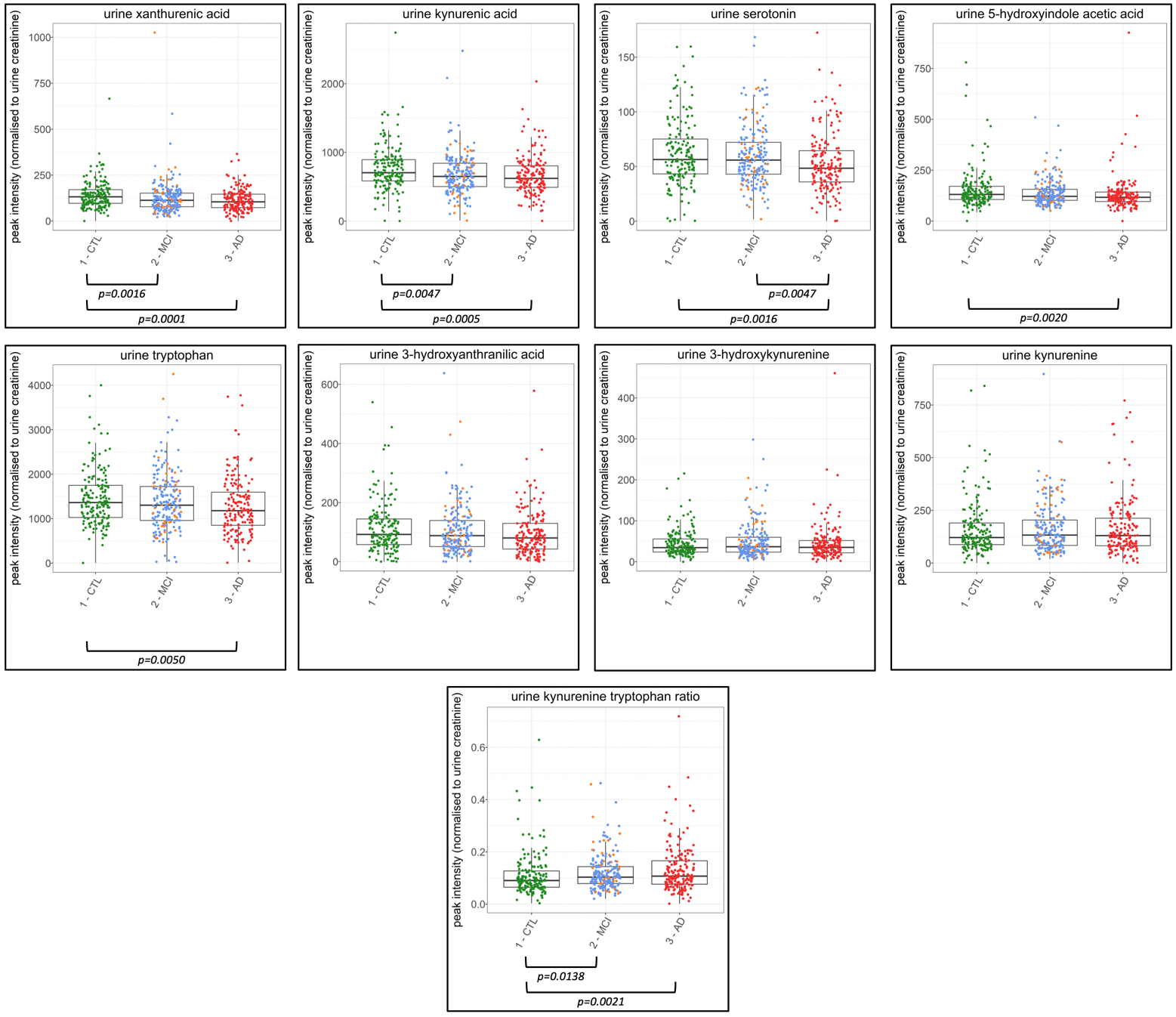
**

**Supplementary Figure S1**

Boxplots highlighting differences between metabolite concentrations in urine when comparing AD (red), MCI (blue = sMCI, yellow = cMCI) and age matched controls (CTL - green). Boxplots are shown for metabolites in the serotonin and kynurenine pathways that were detected in urine. Figure *p* values were calculated using Dunn’s post-hoc test for those metabolites that reported significant inter-group differences in initial Kruskal-Wallis analysis. Significant differences were observed between the participant groups in xanthurenic acid, kynurenic acid, serotonin, 5-hydroxyindole acetic acid, tryptophan and the kynurenine/tryptophan ratio*.*

**
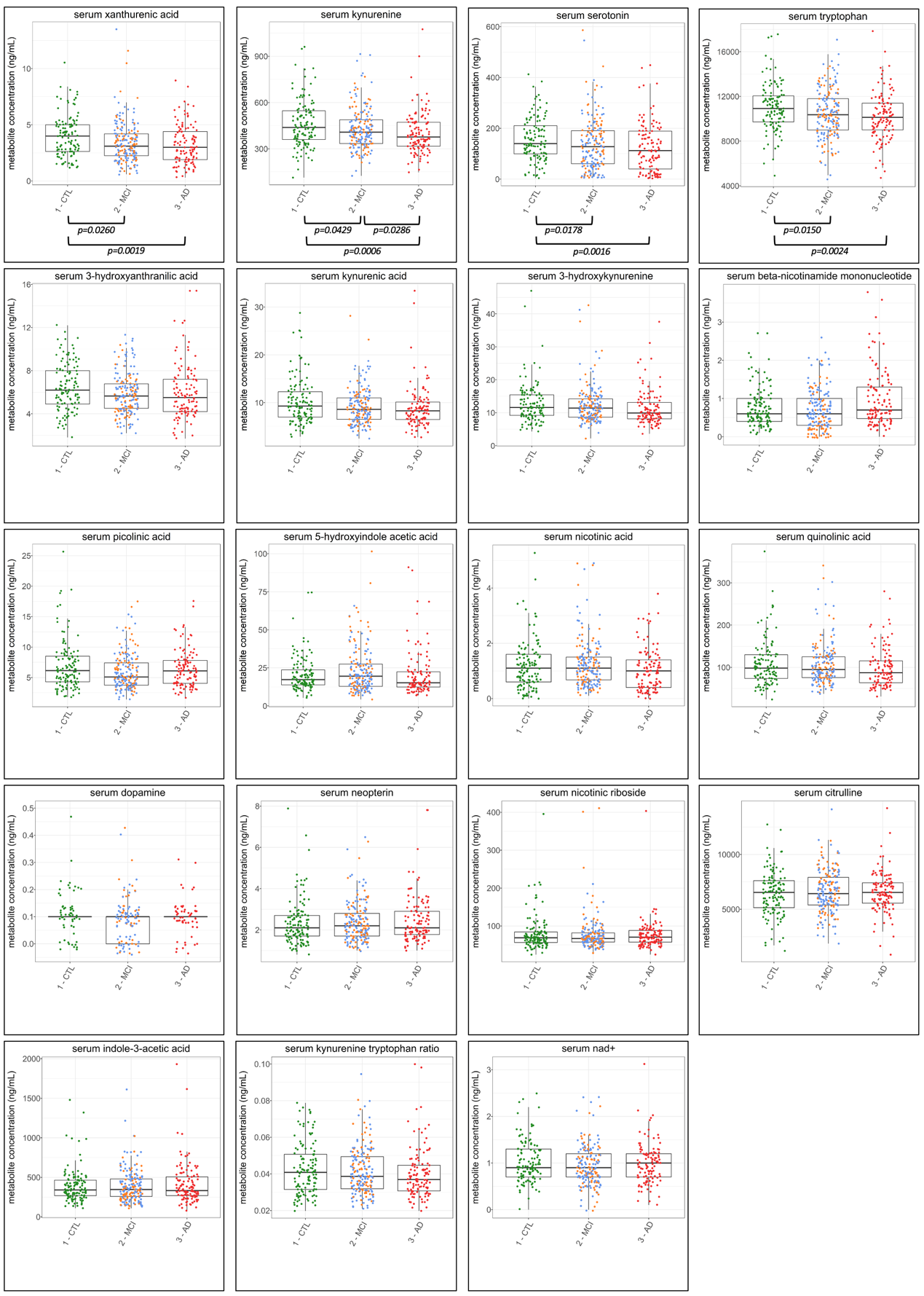
**

**Supplementary Figure S2**

Boxplots highlighting differences between metabolite concentrations in serum when comparing AD (red), MCI (blue = sMCI, yellow = cMCI) and age matched controls (CTL - green). Boxplots are shown for metabolites in the serotonin and kynurenine pathways that were detected in serum. Figure p values were calculated using Dunn’s post-hoc test for those metabolites that reported significant inter-group differences in initial Kruskal-Wallis analysis. Significant differences were observed between the participant groups in xanthurenic acid, kynurenine, serotonin, and tryptophan.
